# Hip hemiarthroplasty through the anterior based muscle sparing approach for femoral neck fractures: an operative technique

**DOI:** 10.3389/fsurg.2025.1673590

**Published:** 2025-11-10

**Authors:** Teddy Cheong, Charles Kon Kam King, Ing How Moo

**Affiliations:** Department of Orthopaedic Surgery, Changi General Hospital, Singapore, Singapore

**Keywords:** hip, femoral neck fracture, hemiarthroplasty, anterior based muscle sparing approach, trauma

## Abstract

Incidence of femoral neck fractures (FNF) in the elderly is rising. Hemiarthroplasty has long been regarded as the mainstay treatment for displaced FNFs in this population. The Anterior Based Muscle Sparing (ABMS) approach for hip arthroplasties is a relatively recent technique which utilises the intermuscular plane between the gluteus medius (GMed) and the tensor fascia lata (TFL) to gain access to the hip joint, thereby sparing the abductor muscles. Due to its proposed benefits of less post-operative pain, faster recovery and lower dislocation rates, it has increased in popularity in recent years. The approach also allows for the safe implantation of any femoral stem design and offers a relatively short learning curve. Given its muscle-sparing nature and favourable stability profile, the ABMS approach is an excellent option for managing displaced FNFs in the elderly population. There is limited literature on the surgical steps of the ABMS approach in hip hemiarthroplasties as treatment for geriatric FNFs. The ABMS approach can be performed in either supine or lateral decubitus position. This article gives a step-by-step description on how to perform a hip hemiarthroplasty using this technique in the lateral decubitus position. Intra-operative videos are provided to illustrate the key points of the surgery.

## Introduction

Femoral neck fracture (FNF) is a debilitating condition with rising incidence and significant morbidity and mortality (1). In the United States, it is estimated that by 2040, the number of hospital admissions for hip fractures will double (2). This population often has decreased bone mineral density and multiple comorbidities which can influence surgical decision-making. Thus, effective management of this condition is of increasing importance. Hemiarthroplasty remains as one of the most common treatment options for displaced FNFs in the elderly population (3). A variety of surgical approaches exist such as the posterior approach, direct lateral approach and the direct anterior approach (DAA) (4).

The Anterior Based Muscle Sparing (ABMS) approach for hip arthroplasties is a relatively recent technique which has increased in popularity in recent years. The ABMS approach was first described by Röttinger in 2004, utilizing the intermuscular interval between the gluteus medius (GMed) and tensor fascia lata (TFL) — commonly known as the Watson–Jones interval — to access the hip for total hip replacement. Therefore, the abductor muscles are not violated (5, 6). Various terminology such as the Rottinger approach, Watson–Jones approach, and anterolateral approach have been used interchangeably to describe this plane. More recently, the term ‘ABMS approach’ has been increasingly adopted in the literature (7–17). ABMS emphasizes its minimally invasive, muscle-sparing philosophy and to distinguish it from the conventional Hardinge (direct lateral) and the DAA. Existing literature has shown the ABMS approach to be effective and safe (7–9). Like the other anterior based approach, the DAA, the ABMS technique offers several benefits including muscle preservation, decreased post-operative pain, faster recovery time and lower dislocation rates (7, 18). However, the ABMS approach utilises a more lateral skin incision away from the inguinal folds as compared to the DAA, where *Cutibacterium avidum* infection rate has been reported to be high due to its incision being close to the groin (19). The ABMS approach has also been reported to have a lower incidence of lateral femoral cutaneous nerve injury as compared to the DAA (10–12). Furthermore, it has a shorter learning curve as compared with the relatively large learning curve associated with DAA (13, 20). Though the ABMS approach has been primarily described as an approach for total hip arthroplasty (THA) (6, 8), there is limited literature on the use of the ABMS approach for hip hemiarthroplasties. This paper aims to minimise the learning curve and optimise effectiveness for fellow surgeons who adopt the increasingly popular ABMS approach by providing a step-by-step description on how to perform a hip hemiarthroplasty using this technique in the lateral decubitus position supplemented with intra-operative videos (Supplementary Video S1–S4) demonstrating key surgical principles.

## Case description

**Figure 1:**
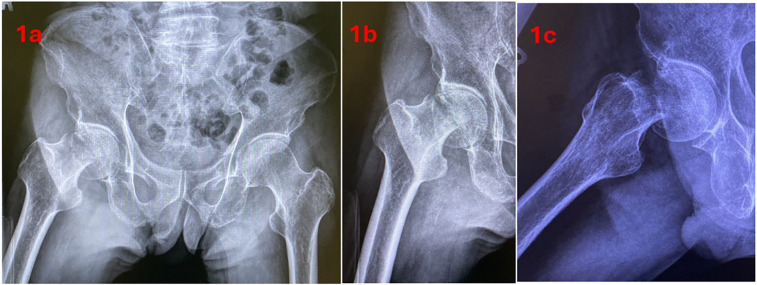
Pre-operative radiographs; **(a)** anteroposterior pelvis view, **(b)** anterior hip view, **(c)** lateral hip view.

The patient involved is an elderly patient who suffered a right hip FNF sustained from a fall. The patient's past medical history consisted of hypertension and Parkinson's Disease. Premorbid, the patient was independently ambulant without the use of a walking aid. Examination revealed a shortened and externally rotated right leg with an intact neurovascular status. Radiographs of the hip demonstrated a displaced subcapital FNF of the right hip (Figure 1). Laboratory investigations were unremarkable. Consent was obtained from the patient for surgical treatment with a hip hemiarthroplasty. Consent was also given by the patient to be included in this article and for video recording of the surgery for the purpose of this article.

## Surgical technique

### Patient positioning and setup

**Figure 2:**
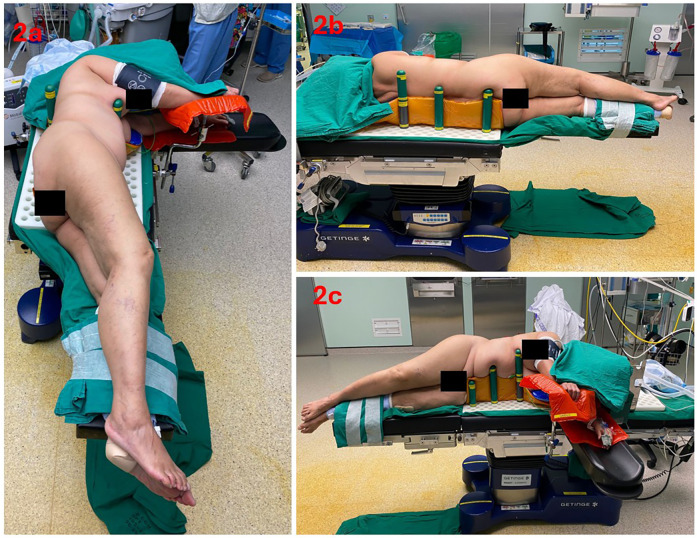
Table set-up and patient positioning. **(a)** lateral view **(b)** posterior view **(c)** anterior view.

This surgery was performed under general anaesthesia. The patient was positioned in the lateral decubitus position on an operating table with a detachable leg plate and peg board. The pelvis was levelled, and the gluteal fold aligned with at the edge of the bed to facilitate hip extension, adduction and external rotation. The contralateral leg was secured to the anterior leg plate in slight hip and knee flexion. Multiple pegs were used in the patient set up. This consists of one short peg placed anterior to the pubis so that the surgeon can palpate the anterior superior iliac spine (ASIS) intraoperatively with unrestricted leg flexion and one long placed at the sacral region. To minimize changes in the patient's position because of intraoperative leg manipulation, the author recommends placing one long peg placed at the anterior and posterior aspect of the upper and lower trunk and a long peg placed anterior and posterior to the thorax. Thus, six pegs were typically used to stabilize the pelvis and trunk. However, the number of pegs can vary according to the size of the patient. The primary surgeon stood at the anterior aspect of the patient (Figure 2).

### Skin incision and identification of the intermuscular interval

**Figure 3:**
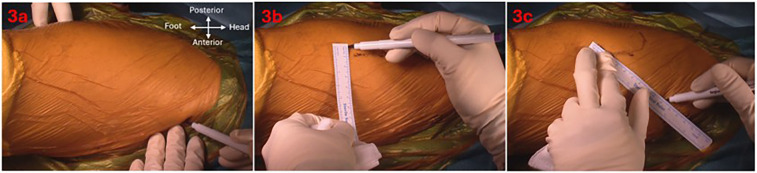
Skin landmarks and incision; **(a)** point A—2 fingerbreadths behind anterosuperior iliac spine, **(b)** point B—5 cm distal to greater trochanter tip and 1 cm posterior to anterior border of femur, **(c)** skin incision from point A to B.

**Figure 4:**
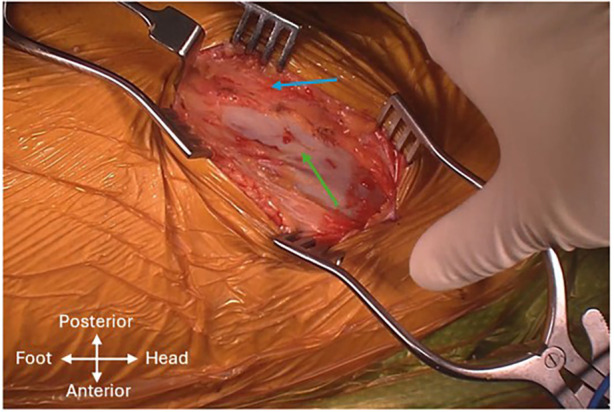
Intermuscular interval with the gluteus medius (blue arrow) and tensor fascia lata (green arrow) seen.

Meticulous skin mapping was performed to guide the skin incision to achieve optimal exposure of the desired intermuscular interval. Landmarks were identified using finger palpation or a 20-gauge spinal needle. Firstly, point A, located two fingerbreadths behind the ASIS was marked. The tip of the greater trochanter (GT) and two points along the anterior border of the femur were marked to outline the femur. Point B, situated 5 cm distal to the tip of the GT and 1 cm posterior to the anterior border of the femur was identified. Points A and B were then connected with an oblique line which corresponds to the anterior border of the GMed, forming the planned incision measuring approximately 10 cm in length. However, this may vary depending on the habitus of each patient (Figure 3). The proximal aspect of the skin incision can be altered based on the preferred femoral stem system. In this case, a broach-only system with an offset handle was used. The incision was deepened with a diathermy until the intermuscular interval is encountered. Care was taken to avoid creating dead space above the fascial layer, minimizing the risk of postoperative seroma formation. The fascia overlying the GMed is thicker and usually appears white, whereas the TFL fascia is thinner and the TFL can appear as a blue hue through the fascia (Figure 4). A linear incision was then made over the GMed, 1 cm posterior to the interval to preserve an adequate fascial cuff for closure at the end of the surgery. The interval was deepened further through blunt finger separation to lift the GMed off the TFL and to avoid inadvertent injury to vessels and nerves (Supplementary Video S1). As the interval is deepened, the terminal transverse branches of the lateral femoral circumflex artery may be seen. Any injury to these vessels should be detected and ligated before proceeding on with the surgery. Proximal dissection of the interval may also reveal the terminal branches of the superior gluteal nerve, which should be preserved. Abduction of the leg can relieve tension and facilitate an easier definition and dissection of the intermuscular plane.

### Capsulotomy and exposure of the fracture site

**Figure 5:**
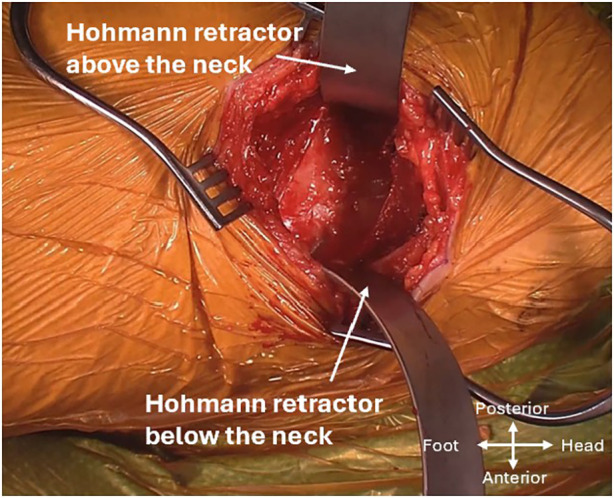
Hohmann retractor placement for exposure of the capsule.

The capsule of the femoral neck will be encountered after going through the intermuscular interval and removal of pericapsular fat. In the context of fractures, these tissue planes may be obscured due to the surrounding haemorrhagic tissue and oedema. Two Hohmann retractors were placed extracapsularly above and below the femoral neck (Figure 5). The leg is externally rotated to allow good exposure of the proximal femur and intertrochanteric ridge. The senior author prefers a Z-shaped capsulotomy, starting from the saddle point of the femoral neck and moving diagonally towards the superior aspect of the acetabulum, followed by another limb moving along the superior rim of the acetabulum and the last limb along the proximal edge of the vastus lateralis moving across the intertrochanteric line. Once this is completed, the extracapsular retractors were placed intracapsularly to reflect the superior and inferior flaps of the capsule off the neck to expose the fracture site and protect posteromedial and anteromedial structures. Upon entry into the hip joint, haemorrhagic fluid may be encountered, which is commonly observed when performing hemiarthroplasty for FNFs (Supplementary Video S2).

### Femoral neck cut and removal of femoral head

Following capsulotomy, the femoral neck was delivered by extending and externally rotating the leg to 90° into a figure of 4 position. With the use of an oscillating saw and osteotome, the femoral neck osteotomy was completed as per the preoperative surgical template plan. Caution was taken during osteotomy to avoid iatrogenic damage to the GT. Fragments of the femoral neck are removed with a rongeur. With the use of a Cobb elevator, the femoral head can be delivered out of the acetabulum with ease. Rarely, a corkscrew drill may be needed to remove the femoral head. The femoral head was then measured (Supplementary Video S2).

### Femur preparation and implant trialling

Adequate femoral exposure is vital to ensure smooth instrumentation. Exposure was obtained with the help of the assistant (standing on the posterior aspect of the patient) by manoeuvring the leg into extension, adduction and external rotation and placed into the sterile pouch (figure of four position). To achieve this, a Hohmann retractor was placed over the posterior aspect of the GT under the abductors and a double-prong retractor was placed at the calcar proximal to the lesser trochanter (LT) to elevate the femur. The double-prong retractor should go in without much resistance. Proper retractor placement is critical to avoid periprosthetic fractures during the surgery, especially in osteoporotic patients. The posterior capsule was sequentially released from the posterior border of the GT. This segment of the surgery requires synergy and coordinated movement between the surgeon and the assistant. The amount of release is tailored based on each patient's size, muscle mass, stiffness and anatomy. In the senior author's experience, in cases of FNFs in elderly patients, release of only the superior capsule is typically sufficient—unlike in osteoarthritic hips undergoing ABMS, where more extensive release of the short external rotators may be required (Supplementary Video S3).

**Figure 6:**
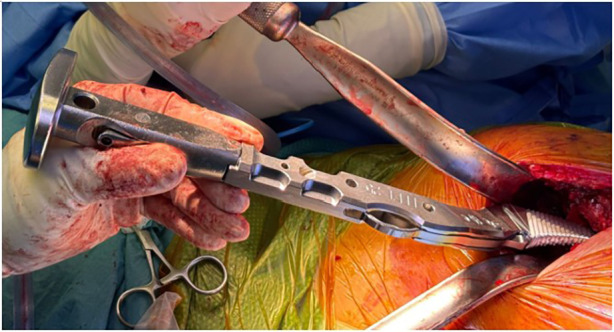
Double-offset handle.

At that point in the surgery, the femur was adequately exposed, and the trajectory of the broach was not hindered by retractors or soft tissues. In the setting of FNF in a geriatric patient, a cemented femoral component is preferred. Box punch was performed and the lateral ridge of bone at the piriformis fossa should be cleared to avoid varus malposition and under sizing of the stem. Remnants of the lateral neck can be further removed with the introduction of a tapered pin reamer down the femoral canal. Broaching was then performed in standard fashion with a double-offset handle (Figure 6). Trial implants are inserted and reduced. Reduction was performed with the leg held in neutral position and longitudinal traction applied by the assistant, while the surgeon lifts the femoral head posterior and lateral over the anterior acetabulum and into the cup. It is important that the surgeon maintains a finger on the trial femoral head and guides it directly into the acetabulum, ensuring that it is well-seated before moving the limb. This prevents the trial head from being displaced by the rectus femoris and lost into the soft tissue. Alternatively, a tagging suture can be placed through the trial head to facilitate retrieval should it become dislodged. Leg length is assessed, and stability is tested via range of motion and shuck test. Once the trial had been tested, it was dislodged by using a bone hook placed around the neck of the stem to lift the femoral head out of the joint while longitudinal traction was applied by the assistant on the leg while in slight abduction (Supplementary Video S3). In the authors’ experience, there was no difficulty during the insertion and extraction of trial implants through the ABMS approach.

### Final implant insertion and closure

Once the component sizes have been selected, fourth generation standard cementing techniques were performed. Standard steps to prepare the femur, not specific to the ABMS, are shown in the video (Supplementary Video S4). This includes the insertion of a cement restrictor, copious irrigation of the femoral canal, thorough drying of the surgical field with the use of gauzes, insertion of the cement followed by pressurisation and eventually the insertion of the final implants and reduction of the hip joint. An Exeter V40 cemented femoral stem (Stryker Orthopaedics, Mahwah, New Jersey, USA) was the implant used in this case. Closure was performed in layers, starting with the capsule and followed by the fascia using vicryl sutures. The skin was closed using Monocryl sutures and sealed with Dermabond Advanced® (Ethicon, Somerville, NJ, USA) (Supplementary Video S4).

## Results

### Post-operative care

**Figure 7:**
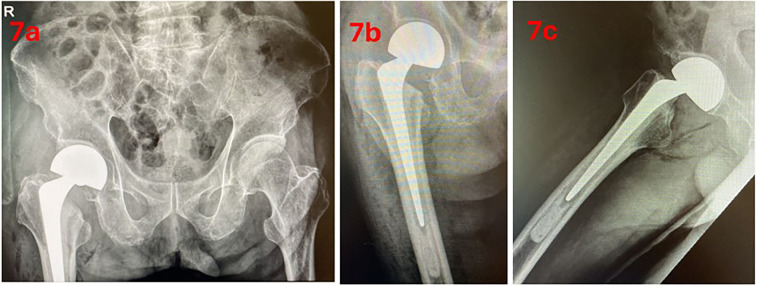
Post-operative radiographs; **(a)** anteroposterior pelvis view, **(b)** anterior hip view, **(c)** lateral hip view.

The patient was allowed to ambulate with full weight-bearing status immediately after the procedure and was commenced on physiotherapy with no hip precautions or restrictions in hip range of motion. Post-operative radiographs were taken (Figure 7). Visual Analogue Score (VAS) was less than two throughout the post-operative period and only received analgesia when needed. The patient received intravenous antibiotics for 24 h and was subsequently discharged well. At the 12-month post-operative mark, the patient was ambulating independently without aid or pain.

## Discussion

The ABMS approach has been shown to be an effective and safe approach (7–9).

### Dislocation rates

Being an anterior based approach to the hip, it has been shown to have low dislocation rates. The dislocation rates in ABMS approach are low and can range from 0% to 0.47% (6, 8, 10, 14). This contrasts with the dislocation rates seen in more traditional approaches. In a study involving 550 patients by Pan et al., they reported a higher dislocation rate in patients who underwent posterolateral approach (3.8%) as compared to those who underwent the ABMS approach (0.47%) (10). Innocenti et al., reported a lower dislocation rate with the ABMS approach (0%) compared to the direct lateral approach (1.5%) in THA (14). Within the anterior approaches to the hip, the ABMS approach and DAA have comparable dislocation rates (10, 12).

### Lateral femoral cutaneous nerve injury

Another advantage of the ABMS is the lower incidence of LFCN injury. LFCN injury is a known complication in DAA as the nerve travels into the proximal thigh, often through the interval between the sartorius and the TFL (21). LFCN injury rates in DAA have been reported to range from 7% to as high as 32% (22–24). The existing literature on the ABMS approach shows a lower rate of reported incidence of LFCN injuries compared to the DAA. In a study by Gorur et al., in New York, they reported that only 1% of patients who underwent THA via the ABMS approach experienced LFCN-associated symptoms, such as numbness, pain or burning sensation (15). Pan et al. compared various surgical approaches for THA and found only 0.94% LFCN injury rate in the ABMS group (10) and Innocenti et al., reported a low LFCN injury rate at 1.4% (14).

### Post-operative pain and mobilisation

The ABMS has been shown to result in less post-operative pain and faster return to mobility compared to traditional approaches. This benefit is likely due to the muscle-sparing nature of the ABMS approach.

Unlike the posterior approach, which often requires strict posterior hip precautions, patients who undergo the ABMS approach typically do not have these restrictions post-operatively. This lack of restrictions is particularly beneficial for elderly patients with conditions like dementia, who may struggle to adhere to post-operative precautions, potentially hindering their ability to ambulate. In Innocenti's study comparing the ABMS and direct lateral approach in THAs, the ABMS group had significantly shorter hospital stay and the timed up and go test, the Harris Hip Score (HHS) and the Oxford hip Score (OHS) were significantly better at the three-month post-operative mark (14). Similarly, George et al. conducted a comparison study between the ABMS and direct lateral approach in THA and found that the ABMS group had significantly lower opioid consumption on postoperative days 1 and 2 and decreased pain intensity during the first 24 h of hospitalisation (25). The ABMS approach and DAA have similar outcomes in this aspect (12, 26, 27). A 2023 meta-analysis comparing the DAA and ABMS approaches found no significant differences in post-operative pain scores and total opioid consumption between the two approaches (12). In a study by Liu et al. comparing the two anterior approaches, Forgotten Joint Scale (FJS-12) scores were significantly higher in the ABMS group compared to the DAA group at two and six weeks postoperatively but the difference at 12 weeks post-operatively was not significant (27).

### Learning curve

An additional advantage of the ABMS is a shorter learning curve. Kagan et al. reported that there was no associated learning curve in their experience in switching from a posterior approach to the ABMS approach. There was no difference in the first 20 patients and each subsequent groups of 20 cases and the ABMS group had a shorter length of stay compared to the posterior approach group (13). Similarly, Nedopil reported a learning curve in transitioning to the ABMS approach to be limited to the first 20 cases (16). In contrast, the DAA has a much steeper learning curve. Peters et al. conducted an analysis of close to 16,000 DAA cases from the Dutch arthroplasty register and found that the learning curve is around 100 cases (20). A systematic review revealed a steep learning curve for the DAA in THA during the first 30 cases and a relative plateau after approximately 100 cases. Operative time reached a relative plateau after approximately 100 cases, suggesting that it takes 100 cases for surgeons to develop proficiency in the DAA (28).

Another advantage of the ABMS approach is its versatility in patient positioning as it can be done in performed in both lateral decubitus and supine positions, whereas the DAA is limited to the supine position.

### Periprosthetic fracture

Despite the benefits of the ABMS approach, there is still the risk of femur-sided complications such as intra-operative fractures of the calcar or GT. In a prospective study in Thailand on hip hemiarthroplasties performed via the ABMS approach, the intra-operative femoral fracture rate was high at 17.5% and was related to the learning curve (the first 11 cases) (17). Innocenti et al. reported a 1.4% rate of intra-operative fracture for the ABMS approach compared with 0% in their direct lateral group (14), while Civinni et al. reported a 0.6% rate of intra-operative fractures in their prospective study (7).

Based on the senior author's experience, this risk can be mitigated by performing adequate soft tissue releases, mainly of the posterior capsule which is done for the purpose of delivering the femur for femur preparation. In cases where exposure proves to be difficult (e.g., large habitus and extensive soft tissue), release of the obturator externus may be necessary. The piriformis and conjoint tendon are preserved. This is similar to the DAA. However, it is important to not excessively release posteriorly as this may increase the risk of posterior dislocation. Accurate and careful placement of retractors and gentle manipulation and broaching of the femur are also very important to minimise this risk of intra-operative fractures.

There is no limitation to the femur stem design with the ABMS approach, but the senior author recommends a cemented stem for FNFs as per international guidelines to decrease the risk of calcar fractures (29). The risk of periprosthetic fracture in the elderly with FNF has been shown to be higher with the use of a cementless stem (26, 30–33). Herndon et al. retrospectively reviewed 684 primary THA performed through the ABMS approach and found that when a cemented stem was utilized, the rate of periprosthetic fracture was 0% compared to 9.8% when cementless stems were used (26). Similarly, Song et al. conducted a study involving 657 cases of bipolar hemiarthroplasties and found a higher rate of periprosthetic fracture in the cementless stem group (3%) as compared with the cemented stem group (0.6%) (30).

Although the learning curve is shorter and lower than the DAA (13, 16, 20, 28), the ABMS approach still requires adequate practice, and the hope of this article is to smoothen the learning curve and minimise complications suffered by other surgeons.

### Strengths and limitations

The strength of this article lies in the detailed description as well as the intra-operative video and pictures provided to illustrate key principles of the surgery to the readers. The aim of the study was to provide a detailed description of the surgical steps involved in performing a hemiarthroplasty through the ABMS approach in a patient in the lateral decubitus position. Therefore, a comprehensive case series/cohort to demonstrate the outcomes of this surgical technique was not provided.

## Conclusion

The ABMS approach is an effective anterior-based approach that offers low dislocation rates, less pain, versatility in positioning and a relatively short learning curve. The key steps and tips described in this article, aided by intra-operative videos, were developed in a hope to minimise the learning curve, reduce intraoperative complications and optimise effectiveness for fellow surgeons who wish to adopt this innovative approach.

## Data Availability

The original contributions presented in the study are included in the article/Supplementary Material, further inquiries can be directed to the corresponding author.

## Supplementary material

Supplementary Video 1 Skin incision and identification of the intermuscular interval.

Supplementary Video 2 Capsulotomy, fracture site exposure, neck cut and head extraction.

Supplementary Video 3 Femur preparation and implant trial.

Supplementary Video 4 Final implant insertion and closure.
